# Fabrication of Nanogroove Arrays on Acrylic Film Using Micro-Embossing Technique

**DOI:** 10.3390/polym15183804

**Published:** 2023-09-18

**Authors:** Chana Raksiri, Potejana Potejanasak, Thitipoom Dokyor

**Affiliations:** 1 Department of Industrial Engineering, Faculty of Engineering, Kasetsart University, Bangkok 10900, Thailand; fengcnr@ku.ac.th; 2 Department of Industrial Engineering, School of Engineering, University of Phayao, Phayao 56000, Thailand; 3 Department of Mechanical Engineering, Faculty of Engineering, Kasetsart University, Bangkok 10900, Thailand; thitipoom.d@ku.th

**Keywords:** micro-embossing process, nanofabrication, polymethyl methacrylate (PMMA) film, nanogrooves

## Abstract

The fabrication of nanostructures is of great importance in producing biomedical devices. Significantly, the nanostructure of the polymeric film has a significant impact on the physical and biophysical behavior of the biomolecules. This study presents an efficient nanofabrication method of nanogroove structures on an acrylic film by the micro-embossing process. In this method, a master mold was made from a thermos oxide silicon substrate using photolithography and etching techniques. An isotropic optical polymethyl methacrylate (PMMA) film is used in the experiment. The acrylic film is known for its excellent optical properties in products such as optical lenses, medical devices, and various general purpose engineering plastics. Then, the micro-embossing process was realized to fabricate nanogroove patterns on an acrylic film by using a micro-embossing machine. However, the morphology of the nanopatterns on an acrylic film was characterized by using an atomic force microscope to measure the dimensions of the nanogroove patterns. The impact of embossing temperature on the morphology of nanogroove patterns on acrylic film is experimentally investigated. The results show that when the embossing temperature is too small, the pattern is not fully formed, and slipping occurs in nanopatterns on the acrylic film. On the other hand, the effect of increasing the embossing temperature on the morphology of nanogrooves agrees with the master mold, and the crests between the nanogrooves form straight edges. It should be noted that the micro-embossing temperature also strongly influences the transferability of nanopatterns on an acrylic film. The technique has great potential for rapidly fabricating nanostructure patterns on acrylic film.

## 1. Introduction

Over the last few years, nanostructures on acrylic film have attracted significant attention owing to their numerous potential applications. Nanostructures on acrylic film have stimulating effects on the physical and biophysical behavior of the cells that adhere to the surface [1]. Various designs of structures on acrylic films, such as nanopillars [2,3,4], nanogrooves [5,6,7], and nanopores [8,9,10] on a nanometer scale have been presented in a wide variety of biomedical devices. Nanostructures on the surfaces of some materials can effectively regulate the adhesion [11,12], migration [13], proliferation [14], and other behaviors of bacteria and viruses. Nanogroove patterns on poly(methyl methacrylate) (PMMA) and polydimethylsiloxane (PDMS) film is a powerful device for increasing cell adhesion properties for medical devices with nanoscale features. Cell elongation, alignment, and proliferation were significantly controlled by the nanogroove-patterned surface [15]. Furthermore, the nanoimprinted polymer solar cell represents a promising and innovative approach in the field of renewable energy and photovoltaics. Through the use of nanoscale patterning techniques, such as nanoimprint lithography, the solar cell’s active layer can be structured with precision, enhancing light absorption and increasing overall device efficiency [16].

A range of conventional fabrication techniques can be used to fabricate a well-defined nanostructure on acrylic film. Nanoimprint lithography (NIL) is a novel method of manufacturing micro/nanometer scale patterns with high throughput and resolution [17,18,19,20]. Nanoimprint lithography relies on an acrylic film’s direct mechanical deformation and achieves solutions beyond the limitations of light diffraction or beam scattering [21,22]. The resolution of NIL mainly depends on the minimum template feature size that can be fabricated [23]. Nanostamps made of silicon dioxide (SiO_2_) contain nanoscale patterns to be fabricated on polymeric materials [24]. When the stamp is filled with a polymer, it is cured by UV light through the stamp, obtaining the stamp’s shape [25,26]. Photolithography with an etching process is the most widely used technique in the nanofabrication of polymeric materials. It involves using an optical technique to produce smaller-scale images, which employs light to transfer a geometric nanopattern from a photomask to a polymeric photoresist. However, this technique has limitations. The resolution of photolithography is limited due to light diffraction. Thus, nanopatterns with a very high resolution, such as sub-hundred nanometers, cannot be prepared on a polymeric photoresist by conventional photolithography. Moreover, these methods require complicated processing steps, aggressive chemistry, and stringent process control, which increase production costs [27,28,29].

The field of nanotechnology has witnessed remarkable advancements, driven by a growing understanding of materials at the nanoscale. Prior research has explored various techniques for the precise control and manipulation of metallic structures at this level. For instance, the use of the chemical lift-off process [30,31,32,33,34] has shown promise in the rapid and cost-effective fabrication of metallic nanostructures. This technique, while innovative, has prompted further investigation into its scalability and application in the real world. Furthermore, the fabrication of metallic nanodot arrays has been explored using the nanochemical stamping technique with a polymer stamp [35,36]. This approach offers a high degree of precision and repeatability, making it suitable for applications in nanoelectronics and photonics. However, challenges remain in terms of stamp durability and pattern transfer fidelity. These methods contribute significantly to the development of advanced materials and devices, providing valuable insights into the fabrication of metallic nano/microstructures for various scientific and technological applications. Further exploration and refinement of these techniques hold the promise of unlocking even more exciting possibilities in the realm of nanoscale materials and devices.

To overcome the limitation of conventional nanofabrication methods, we have recently developed the micro-embossing technique that can fabricate nanopatterns on an acrylic film. This study of fabrication techniques of a polymer film was conducted experimentally. Experiment works were conducted to study the suitable conditions for fabricating nanogroove patterns on an acrylic thin film. A mother mold was made from a silicon wafer. Then, a polymer film mold was fabricated using the simple micro-embossing process. Experiments on various kinds of hot imprinting techniques were performed to obtain the optimum hot imprinting process, which provides the high transferability of nanopatterns on an acrylic film.

## 2. Materials and Methods

### 2.1. Polymethyl Methacrylate (PMMA) Film

Acrylic films, also known as polymethyl methacrylate (PMMA) film, are thermoplastics known to be some of the most economical, easily fabricated films. Acrylic films are attractive materials for realizing fabrication devices in various applications, such as anti-reflection film, optics, and micro lenses. Their fast and easy processing technologies allow for cost-effective mass production, while their tunable properties provide high flexibility in design. The acrylic film used in this study was Acryplen-HBA002P, Mitsubishi Chemical Engineering Corporation (Nagahama-shi, Japan), and features excellent transparency, weatherability, and suitability for heat-molding and lamination processing. In addition, it has outstanding ultraviolet protection, making it ideal for use as a construction material, a paint alternative, and a retro-reflective material, as well as for a wide range of applications in the field of optical engineering, medical devices, and various general purpose engineering plastics. Acryplen exhibits the superior optical qualities of high transparency with a total transmission of 92.6%, excellent weatherability, and excellent precision moldability [37]. Acryplen features outstanding molding processability. The glass transition temperature (*Tg*) of Acryplen is about 90 °C. Since Acryplen exhibits high fluidity and facilities the precise transferring of sub-micro patterns, a more comprehensive range application is, therefore, being developed. In this study, the nanopatterns were fabricated on acrylic film, 100 and 188 µm of thickness, using the micro-embossing technique. Typical properties of Acryplen are soft type transparency weatherability, as illustrated in Table 1.

### 2.2. Fabrication of a Master Mold with Nanostructure Patterns

**Figure 1:**
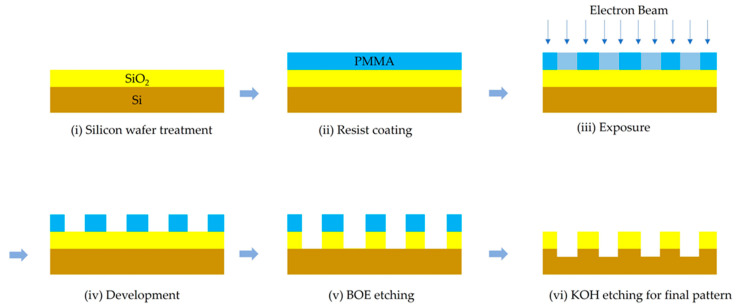
Electron beam lithography and etching technique to fabricate a master mold.

A master mold was made from a thermos oxide silicon substrate. The silicon wafer <100> is the best material to use as a master mold. It is easy to process and allows for imprinting all kinds of nanometer patterns with high aspect ratios. A series of parallel nanogroove arrays were fabricated using photolithography and etching techniques. The nanofabrication process of a silicon wafer master mold is shown in Figure 1.

(i) Silicon wafer was cleaned to remove the surface contamination. Silicon wafer was cleaned with 150 mL of boiled sulfuric acid at 100 °C, 150 mL of hydrogen peroxide as a catalyst. Then, silicon wafer was washed with reverse osmosis water (RO water) to remove sulfuric acid and hydrogen peroxide. However, silicon wafer was immersed in nitric acid to treat the surface of the oxide layer. Then, wafer was dried with nitrogen gas and dehydrated in an oven at 150 °C for about 4 h. Furthermore, silicon wafer was cleaned using plasma with oxygen gas at 300 watts for 20 min, resulting in the growth of a thin layer of silicon dioxide (SiO_2_) on the surface. Furthermore, organic compound on silicon wafer was removed;
(ii) The polymethyl methacrylate, PMMA 950K-A2 (2% PMMA molecular weight of 950,000 g·mol^−1^) was diluted in anisole. Anisole was used as a solvent for PMMA due to its ability to dissolve the polymer effectively. The dilution ratio was 1:1. Then, PMMA–anisole solution was coated on silicon wafer via spinning process. On the first layer, PMMA was spin-coated onto the surface of silicon wafer at 500 rpm for 10 s. Then, the second layer was operated at 1500 rpm for 40 s to increase the thickness of PMMA. The spun wafer was heated at 180 °C with heating time around 5 min to achieve the 80 nm thickness of photoresist film;
(iii) In order to be exposed to the electron beam lithography process, the TESCAN MIRA3 machine was utilized. The silicon wafer was put inside the chamber and the pressure was reduced zero. The beam current was about 0.216 nA, and the exposure dose at 400 μC/cm^3^. The micro-grooves pattern was fabricated onto the surface of PMMA on silicon wafer. The distance between peak to peak of nanogroove arrays was about 500 nm, around 2.5 × 2.5 cm^2^. The exposed photoresistance experienced chemical changes by the electron beam, achieving polymer scission and breaking the polymer chain;
(iv) The specimen was developed in methyl isobutyl ketone:isopropyl alcohol solution (MIBK:IPA, 3:1) for 50 s. An exposed polymer chain was washed up. A masking layer was prepared on the specimen for etching in the following process;
(v) Buffered oxide etch (BOE) process was used to remove SiO_2_. BOE is a very selective etch that stops at the silicon and does not etch further. In this process, 400 g of ammonium fluoride (NH_3_F) with 600 mL of DI water (DIH_2_O) was mixed. Then, ammonium fluoride solution was mixed with hydrofluoric acid (HF) in a 6:1 ratio. The solution was utilized to remove an oxide layer on a master mold. Silicon wafer master mold was immersed in the solution for 140 s. A result shows that the unmasked oxide layer is exposed as nanogroove arrays are apparent on silicon wafer master mold.
(vi) The wet chemical etching process by potassium hydroxide (KOH) etching was used to create cavities as nanogroove arrays on silicon wafer. A solution of potassium hydroxide at 40% concentration was prepared and heated to 60 °C. Silicon wafer was immersed into a solution for 10 min to remove a resistant out-of-surface silicon wafer. Finally, a master mold was washed up with hydrochloric acid to adjust the pH value.

### 2.3. Micro-Embossing Process

**Figure 2:**
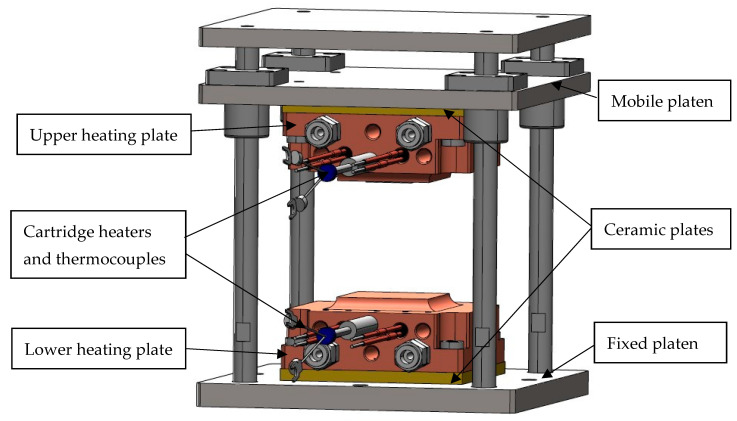
Schematic drawing of micro-embossing machine.

The micro-embossing process was used to fabricate nanopattern arrays on an acrylic film. The micro-embossing process was realized on a micro-embossing machine. Figure 2 illustrates the schematic diagram of the micro-embossing device using SolidWorks version 2018. It has an upper and lower heating plate made of copper. The upper heating plate was fastened with bolts on the mobile platen, and the lower plate was fastened with bolts on the fixed platen. The ceramic vessels were used to protect the components from the effects of heat. The cartridge heaters and thermocouples were inserted into the upper and lower heating plate and connected to the temperature controller. The digital temperature controller for heaters (Digital fine thermo, DG2-SSR, 100VAC-220VAC, Hakko Electric Co., Ltd., Hakusan-shi, Japan) was used to control the heating temperature of the upper and lower heating plate.

**Figure 3:**
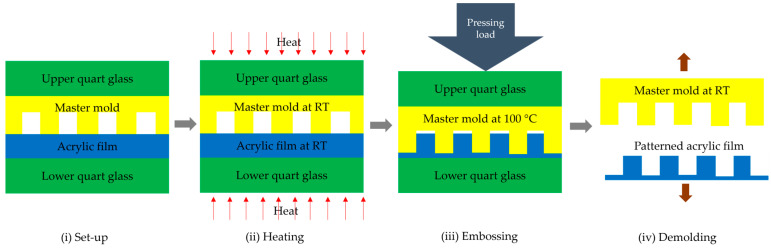
Schematic illustration of the micro-embossing method to fabricate nanogroove structures on an acrylic film.

Figure 3 illustrates the micro-embossing processes to fabricate nanogroove arrays on an acrylic film. The process is comprised of four steps.

**Figure 4:**
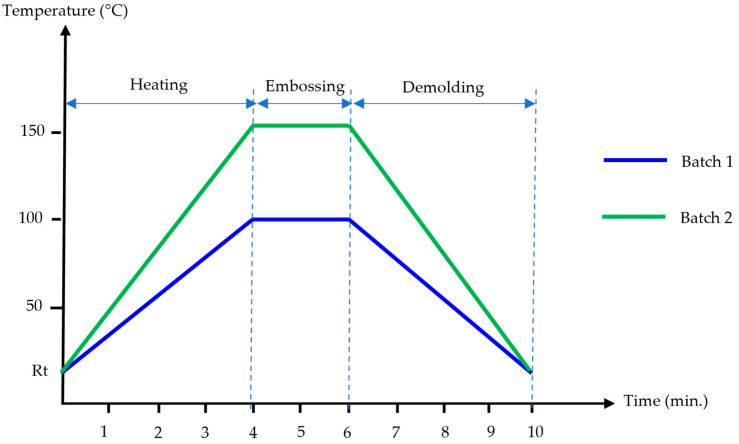
Processing curve of micro-embossing process.

(i) The set-up for experiment testing was prepared. The size of the upper and lower square quartz glass substrates was 1.2 mm × 20 mm × 20 mm. The lower quartz glass substrate was inserted below a master mold. An acrylic film was placed upon a master mold. The size of the acrylic film was 20 × 20 mm^2^, and the thickness was 100 and 188 µm. Another quartz glass substrate was placed upon an acrylic film to prevent stress concentration at the upper cylinder that caused the breakage of the master mold;
(ii) In the heating stage, the set-up of an acrylic film and master mold was placed in the micro-embossing machine. They were heated from room temperature (RT) to 100 °C and 150 °C. The heating time was about 4 min;
(iii) In the embossing stage, a pressing load was applied by pressing the upper cylinder down on the quartz glass substrate. The weight of the upper heating plate and the mobile plate was about 10 kg. The embossing pressure was 0.1569 MPa onto the set-up of an acrylic film and master mold. The glass transition temperature (*Tg*) of the Acryplen film was 90 °C. The micro-embossing process was carried out above an Acryplen’s glass transition temperature at 100 °C and 150 °C, as shown in Figure 4. Then, the embossing time was kept for 2 min. The flow behavior of acrylic film can be enhanced by changing temperature and pressure. The upper cylinder was manually hiked, and the specimen was taken out of the micro-embossing machine;
(iv) In the demolding stage, the specimen was turned out of the micro-embossing machine and cooled down at room temperature for 4 min. The unwinding process for an acrylic film at room temperature allows easy film removal and prevents master mold breakage. Then, an embossed acrylic film was peeled by using tweezers. Thus, an acrylic film with nanostructure was successfully fabricated. In addition, the total cycle time of the micro-embossing process was about 10 min. Finally, the protuberances of micropattern arrays on a stamped acrylic film were analyzed by atomic force microscope.

## 3. Results

### 3.1. Nanostructure on a Master Mold

**Figure 5:**
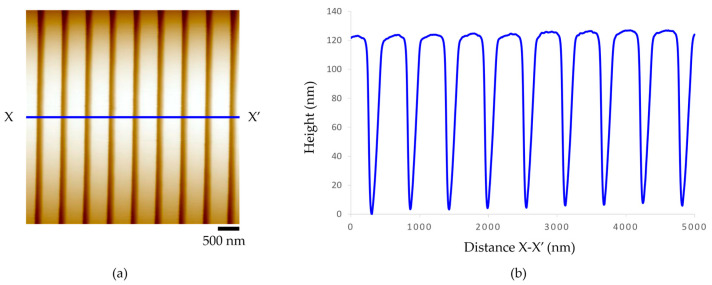
Nanogroove patterns on a master mold (**a**) AFM image and (**b**) height profile.

The morphology of nanopatterns on a master mold was analyzed using atomic force microscopy (AFM, Keyence VN-8010, Keyence Co., Ltd., Tokyo, Japan) to measure the dimensions of nanopatterns. The result shows that the average distance from peak to peak of the nanogroove arrays was about 500 nm, the average width of the groove about 300 nm, and the average height about 120 nm, as shown in Figure 5. Nanogroove patterns on the silicon wafer mother mold were used to fabricate the nanogroove structures on an acrylic film.

### 3.2. Fabrication Quality of the Micro-Embossing Process of the Acrylic Film with Nanostructures

To study the process conditions of the micro-embossing process of acrylic films and to verify the quality of the fabricated nanostructure patterns, we conducted experiments. The experiment of this study was categorized into two batches. In the first batch, the acrylic film micro-embossing process with the embossing load and time was maintained at 0.1569 MPa and 2 min. Then, the practical embossing temperature was found to be 100 °C, as shown in the blue line in Figure 4. The morphology of the nanogroove pattern on an acrylic film was characterized using an atomic force microscope (AFM, Keyence VN-8010) to measure the dimensions of the patterns. 

**Figure 6:**
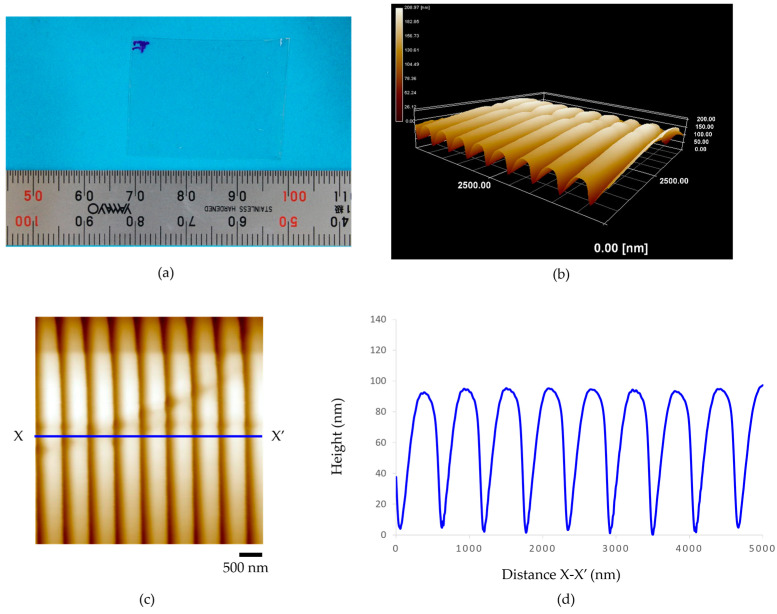
The 100-µm-thickness acrylic film of micro-embossing process at embossing load for 0.1569 MPa, embossing time for 2 min, and embossing temperature of 100 °C: (**a**) an acrylic film after fabrication, (**b**) the three-dimensional AFM topography image, (**c**) the AFM topography image, and (**d**) height profile.

**Figure 7:**
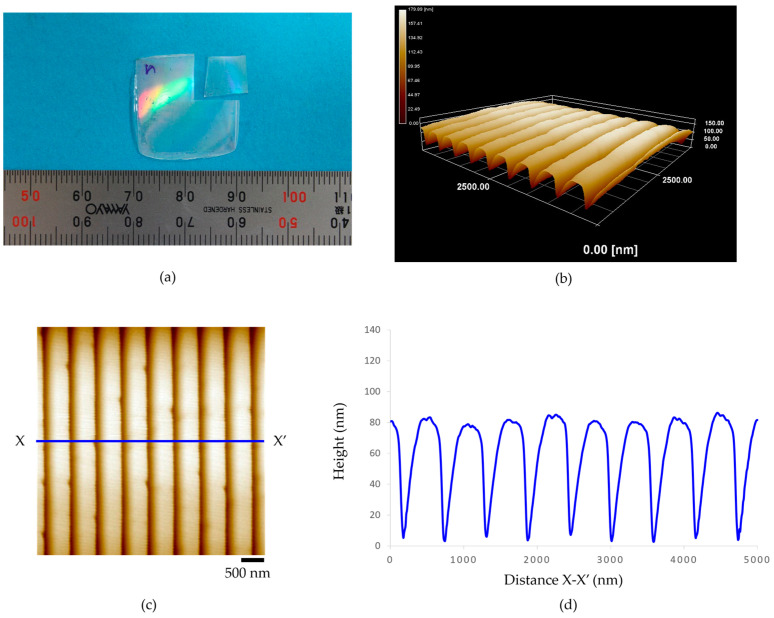
The 188-µm-thickness acrylic film of micro-embossing process at embossing load for 0.1569 MPa, embossing time for 2 min, and embossing temperature of 100 °C: (**a**) an acrylic film after fabrication, (**b**) the three-dimensional AFM topography image, (**c**) the AFM topography image, and (**d**) height profile.

Figure 6 presents (a) a 100-µm-thickness acrylic film of the micro-embossing process, (b) the three-dimensional AFM topography image, and (c,d) illustrate the AFM topography image and height profile. The experimental results show that the patterns of nanogroove structures are uniformly transferred onto the surface of the acrylic film. It was also confirmed that the average distance between the peaks of the grooves was about 510 nm, the average width of grooves was about 200 nm, and the average height of the nanogrooves was approximately 85 nm. However, horizontal collapses were found across the alignment of the nanogroove patterns. Additionally, Figure 7 illustrates the experimental results of (a) the micro-embossing of a 188-µm-thickness acrylic film. The average distance between the peaks of the nanogrooves was about 580 nm, the average width of the nanogrooves was about 205 nm, with the average height being about 80 nm, as shown in (b–d). As seen in (c), many surface defects were observed. The small notches were found at the tip of the nanogroove patterns. 

The experimental results of the first batch directly prove that the embossing temperature at 100 °C was inapplicable for the micro-embossing process. Defects were found on the surface of the acrylic films after the embossing step. It should be noted that the embossing temperature also strongly influences the transferability of nanogroove patterns on an acrylic film. To overcome this problem, the process conditions of the micro-embossing process were modified for the second batch. Then, the practical embossing temperature was found, as shown in the green line of Figure 4. The embossing temperature of 150 °C was investigated experimentally.

**Figure 8:**
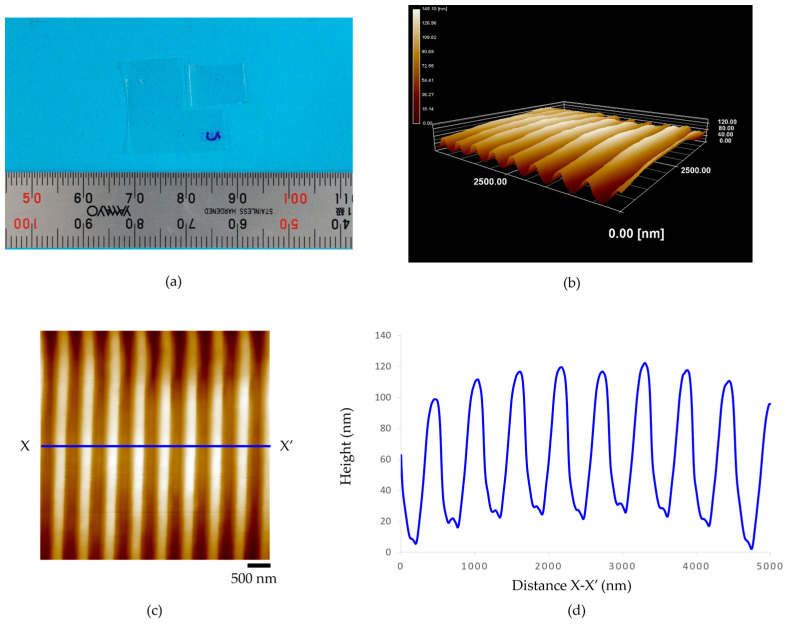
The 100-µm-thickness acrylic film of micro-embossing process at embossing load for 0.1569 MPa, embossing time for 2 min, and embossing temperature of 150 °C: (**a**) an acrylic film after fabrication, (**b**) the three-dimensional AFM topography image, (**c**) the AFM topography image, and (**d**) height profile.

**Figure 9:**
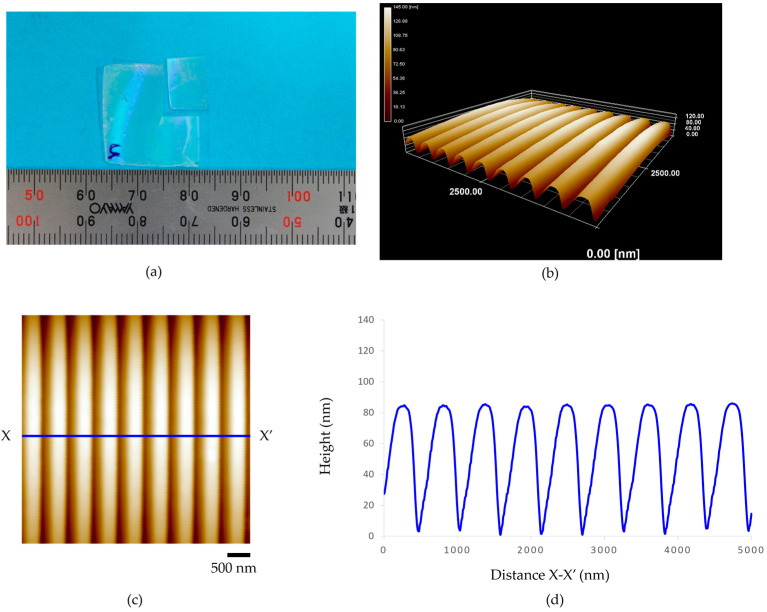
The 188-µm-thickness acrylic film of micro-embossing process at embossing load for 0.1569 MPa, embossing time for 2 min, and embossing temperature of 150 °C: (**a**) an acrylic film after fabrication, (**b**) the three-dimensional AFM topography image, (**c**) the AFM topography image, and (**d**) height profile.

The morphology of the nanogroove patterns on a 100-µm-thickness acrylic film after the micro-embossing process at 150 °C was characterized, as shown in Figure 8a. There, (b) presents a three-dimensional AFM topography image, (c) illustrates a top view of the nanogroove pattern, and (d) height profile. The results show that the series of parallel grooves are shown to be transferred onto the surface of a 100-µm-thickness acrylic film. It was also revealed that the average distance between the peak of the nanogrooves was approximately 500 nm, the average height of the grooves was approximately 105 nm, and the average width of the grooves was about 180 nm. In addition, the morphology of the nanogroove patterns on a 188-µm-thickness acrylic film after the micro-embossing process at 150 °C was characterized, as shown in Figure 9: (a) shows the micro-embossing of a 188-µm-thickness acrylic film. Then, (b) presents a three-dimensional AFM topography image, (c) illustrates a top view of the nanogroove pattern, and (d) height profile. The results show that the series of parallel grooves are transferred onto the surface of a 188-µm-thickness acrylic film. It was also revealed that the average distance between the peak of the nanogrooves was approximately 510 nm, the average height of the grooves was about 87 nm, and the average width of the grooves was about 200 nm. It was also observed that the crests between the grooves formed straight edges. The slipping of nanogroove patterns did not occur.

## 4. Discussion

Micro-embossing is a conventional nanofabrication process that combines pressing with heating to transfer nanopatterns from a master mold to another material [41,42,43]. However, this method has weaknesses, such as applying an excessive pressing load will cause master mold to break [44,45]. In addition, overheating at the embossing step may cause deformation of the acrylic thin film [46,47]. Furthermore, pulling an embossed acrylic film out of a master mold may cause damage to the nanogrooves on a master mold [48]. Therefore, this study attempted to find the appropriate micro-embossing process for fabricating the nanogrooves on an acrylic film without damaging the silicon wafer master mold and acrylic thin film. 

A silicon wafer master mold was prepared that has pointed top edges for the nanogrooves pattern. When the master mold was placed on an acrylic thin film and the temperature was raised above the glass transition temperature, the wedge of nanogrooves helped the master mold sink into the polymer melt with the pressing load of the upper heating plate and the mobile plate of the micro-embossing machine. The embossing temperature was identified as the critical parameter to obtain nanometer-sized replicas on an acrylic film [49,50,51]. Therefore, this study determined time and embossing pressure at a constant level in every batch of experiments. In brief, we found that compared to the embossing temperature near a glass transition temperature (*Tg*) of Acryplen film at 100 °C, the embossing temperature at 150 °C (above *Tg* about 60 degrees) ensures a more accurate replica geometry on an acrylic film. As the temperature rises, the viscosity of an acrylic film decreases [52], which allows the melt of an acrylic film to flow faster into the master mold cavities. Therefore, the fluidity of a melted acrylic film must be enhanced by increasing the embossing temperature [53]. These results indicate that an acrylic film does not flow into the nanometer cavities at temperatures near *Tg*, and the optimum embossing temperature of this study appears to be at 60 °C above *Tg*. 

**Figure 10:**
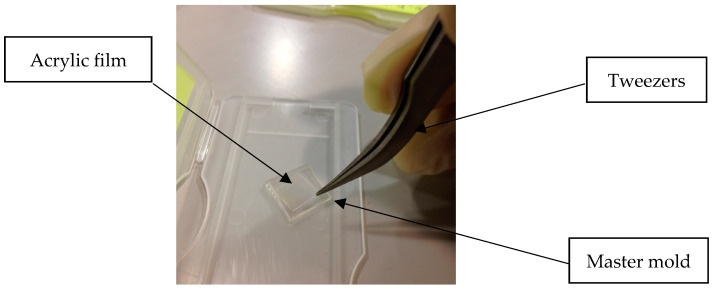
The demolding stage of micro-embossing process by using the nib tweezers at room temperature.

In this study, the micro-embossing process requires using master mold for replication. However, the fragile nanogrooves on the master mold were easily damaged during the demolding stage [54,55]. Fractures that occur in the nanopattern of the master mold mean obtaining an incomplete copy of the nanogrooves on acrylic film. Therefore, an embossed acrylic film in the master mold was turned out of the micro-embossing machine after the embossing step and cooled to room temperature. Then, an embossed acrylic film was carefully peeled out of a master mold using the nib tweezers, as shown in Figure 10. The result shows that an embossed acrylic film was easily removed from the master mold.

**Figure 11:**
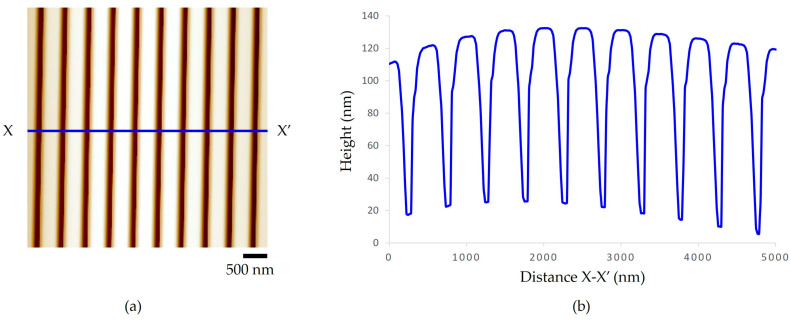
Nanogroove patterns on a master mold after micro-embossing process (**a**) AFM image and (**b**) height profile.

Furthermore, after each micro-embossing experiment, the nanopatterns on the master mold were analyzed by AFM. The results show that the average distance of the nanogroove arrays from peak to peak was about 500 nm, the average width of the groove was about 300 nm, and the average height was about 120 nm, as shown in Figure 11. It was confirmed that the nanogroove structures on the silicon wafer master mold still have the same dimensions as before the fabricating process. It was also revealed that the nanogroove pattern are still in the appropriate dimension for replicating the micro-embossing process. 

While the material advantages of utilizing acrylic film in the fabrication of nanogroove arrays are certainly noteworthy, it is essential to redirect attention towards the significant technical advantages that the micro-embossing technique offers in this process. This approach not only enhances precision and repeatability but also enables the creation of intricate and tailored nanostructures with remarkable efficiency. As this process uses an embossing temperature that is not too high, it does not take long to press with a suitable pressure. Therefore, the master mold in this study can be reused more than 100 times without breaking. By emphasizing these technical aspects, we can gain a deeper understanding of the true potential and innovations that arise from the application of micro-embossing in nanogroove array fabrication.

Another method that can protect mold damage is to use a soft thermal mold imprinting method. The soft thermal nanoimprint technique mold with a 10 nm feature size represents a significant advancement in nanofabrication technology. The method’s ability to create high-resolution patterns on various substrates with improved replication fidelity and reduced defects is highly promising for applications in microelectronics, photonics, and nanotechnology. By using a soft and flexible PDMS mold, the process minimizes damage to delicate structures and enables the fabrication of complex patterns with superior precision. Moreover, the low-cost and high-throughput nature of the soft thermal nanoimprint process makes it a compelling alternative to conventional lithography techniques [56].

## 5. Conclusions

This study presents the micro-embossing of acrylic films with nanogrooves. The mother mold is prepared from silicon wafer. Silicon wafer is a suitable material to use as a master mold as it is easy to process and allows the fabrication of all kinds of nanometer patterns with a high aspect ratio. The photolithography and etching technique fabricated the nanogroove patterns on the silicon wafer mother mold. Then, the micro-embossing approach used the silicon wafer master mold to transfer the nanogroove structures to an acrylic film. This study attempted to find the appropriate micro-embossing condition for fabricating the nanogroove structures on an acrylic film without damaging the silicon wafer master mold and acrylic thin film. The results show that the micro-embossing process fabricated nanogroove patterns at an embossing load of 0.1569 MPa, with an embossing time of 2 min, and an embossing temperature of 150 °C. The distance between peaks of neighboring grooves on an embossed acrylic film agrees with the spread of parallel nanogrooves on the silicon wafer master mold. The crests between the nanogrooves formed straight edges. The nanogrooves on the master mold were still in good condition for the replicating process. Furthermore, the throughput time of this process was about 10 min per specimen. Most importantly, large-area nanostructure patterns were successfully fabricated on an acrylic film through a single-step micro-embossing process with high throughput. The entire fabrication process, from creating the silicon wafer master mold to transferring the nanogroove structures onto the acrylic film is accomplished through a single-step micro-embossing process. This simplicity and directness of the method contribute to its practicality and potential for industrial adoption. The technique offers high replication fidelity, minimal damage to materials, and a single-step process, making it a valuable approach for large-scale production of nanoscale patterns for various applications in electronics, optics, and other fields.

## Figures and Tables

**Table 1:** ACRYPLEN general properties.

Properties	Methods	Unit
Total transmittance	JIS K7361-1 [38]	92.6%
haze	JIS K7136 [39]	0.9%
Heat shrinkage	MCC Internal (100 °C, 10 min)	MD = 11.4% TD = −1.1%
*Tg* (DSC)	MCC Internal	90 °C
Tensile strength	JIS K7127 [40]	MD = 34 MPa TD = 33 MPa
Elongation	JIS K7127 [40]	MD = 138% TD = 136%
Chemical resistance	Acid (0.1 N H_2_SO_4_)	Stable
Chemical resistance	Alkali (0.1 N NaOH)	Stable
Chemical resistance	Petroleum chemical (acetone, ethyl acetate)	Dissolved
Chemical resistance	Petroleum chemicals (methanol)	Liquid mark

MCC internal method with reference to ISO or JIS.

## Data Availability

Not applicable.
